# The HOMA-IR Performance to Identify New Diabetes Cases by Degree of Urbanization and Altitude in Peru: The CRONICAS Cohort Study

**DOI:** 10.1155/2018/7434918

**Published:** 2018-12-16

**Authors:** Rodrigo M. Carrillo-Larco, J. Jaime Miranda, Robert H. Gilman, William Checkley, Liam Smeeth, Antonio Bernabe-Ortiz, CRONICAS Cohort Study Group

**Affiliations:** ^1^CRONICAS Center of Excellence in Chronic Diseases, Universidad Peruana Cayetano Heredia, Lima, Peru; ^2^Department of Epidemiology and Biostatistics, School of Public Health, Imperial College London, London, UK; ^3^Department of Medicine, School of Medicine, Universidad Peruana Cayetano Heredia, Lima, Peru; ^4^Department of International Health, Bloomberg School of Public Health, Johns Hopkins University, Baltimore, USA; ^5^Área de Investigación y Desarrollo, AB PRISMA, Lima, Peru; ^6^Division of Pulmonary and Critical Care, School of Medicine, Johns Hopkins University, Baltimore, USA; ^7^Faculty of Epidemiology and Population Health, London School of Hygiene and Tropical Medicine, London, UK

## Abstract

**Aims:**

Prognostic thresholds to identify new type 2 diabetes mellitus (T2DM) cases using the HOMA-IR have not been defined. We studied the HOMA-IR performance to identify incident T2DM cases and to assess if the thresholds varied according to urbanization and altitude in Peru.

**Methods:**

Longitudinal analysis. The outcome was incident T2DM cases: self-report diagnosis and fasting glucose. The exposure was the HOMA-IR. Receiver operating characteristic (ROC) curves were plotted, and the area under the ROC curve (AUC) was estimated with 95% confidence intervals (95% CIs). Results are presented overall and stratified by study site (Lima, Tumbes, urban Puno, and rural Puno), rurality (urban, semiurban, and rural), and altitude (low and high).

**Results:**

A total of 3120 participants (mean age: 55.6 years, 51.2% females) contributed data to this analysis. The median baseline HOMA-IR was 1.7 (IQR 1.0–2.9), with median values ranging from 1.1 in rural Puno to 2.0 in Lima and Tumbes (*p* < 0.001). Overall for incident T2DM, the AUC was 0.69 (95% CI: 0.64–0.74) with an empirical threshold of 2.8 yielding a positive likelihood ratio of 2.30 and a negative one of 0.61; the positive and negative predictive values were 14.6% and 95.7%, respectively. The empirical thresholds varied within the variables of interest, for example, from 0.9 in urban Puno to 2.9 in Lima.

**Conclusions:**

Using the HOMA-IR to identify incident T2DM cases seems to yield moderate accuracy. The HOMA-IR could help improve identifying people at high risk of T2DM.

## 1. Introduction

Type 2 diabetes mellitus (T2DM) has become one of the largest health problems in the world. It is among the top causes of disability-adjusted life years as well as years lived with disability, particularly in middle- and high-income countries [1, 2]. The scenario is not different in Peru, where population-based studies in several areas reported a 7% prevalence [3, 4] and an incidence of two new cases per one hundred person-years [4, 5]. These international and national figures pinpoint the value of T2DM prevention and early diagnosis.

An important approach to prevent or delay the T2DM burden is to identify those individuals at high risk of T2DM. Therefore, tools to identify T2DM cases, taking advantage of available resources and which are reliable in a target population, are needed. Laboratory facilities, along with their processes and logistics, are known by health systems in low- and middle-income countries, even though they may not be available in all health facilities. Identifying laboratory-based tests for early identification of T2DM may be an important asset and can potentiate the use of available resources instead of introducing completely new approaches. In this line, the homeostasis model assessment for insulin resistance (HOMA-IR), which combines fasting glucose and insulin, could be an option [6–10]. The HOMA-IR assesses the function of *β*-cells and thus informs physiological changes that occur before the onset of T2DM (i.e., precursor of T2DM) [11, 12], which makes this test a potential tool to identify people at high risk of developing T2DM in the near future. In addition, HOMA-IR thresholds to identify T2DM cases have not been established, and neither have they been studied in populations living in places at different stages of urbanization or altitude above the sea level. Consequently, this study is aimed at determining the HOMA-IR performance to identify incident T2DM cases in four areas in Peru. This study is aimed at providing evidence to further use existing laboratory tests—insulin and glucose—that could be available in health facilities.

## 2. Methods

### 2.1. Study Design

This is a longitudinal analysis using data of the CRONICAS Cohort Study [13]. Briefly, the CRONICAS Cohort Study is a population-based prospective cohort which started in September 2010. To date, two follow-up rounds have been conducted, fifteen and thirty months, on average, after the baseline recruitment.

### 2.2. Study Scenario

The CRONICAS Cohort Study was conducted in four Peruvian areas: Lima, Tumbes, rural Puno, and urban Puno [13]. These sites have different socioeconomic and geographical features. Lima is a highly urbanized metropolis at sea level. Tumbes is a semiurban area at sea level. Puno is a city at 3825 meters above the sea level, from which two different sites—urban and rural—were studied.

National statistics rank Lima with the highest human development index (0.63), followed by Tumbes (0.52) and then Puno (0.39) [14]. The 62.6% of the population in Lima had any health insurance, and this figure for Tumbes and Puno was 68.7% and 60.7%, respectively [15]. The illiteracy frequency for people aged ≥15 years in these settings was 2.3% in Lima, 3.0% in Tumbes, and 9.4% in Puno [16], whereas life expectancy was 77 years in Lima, 74 years in Tumbes, and 70 years in Puno [17].

### 2.3. Study Population

At baseline, the CRONICAS Cohort Study aimed for a sex- and age- (35–44, 45–54, 55–64, and ≥65 years) stratified random sample of at least 1000 people in each study setting (500 in urban and rural Puno) [13]. An updated census was used to identify eligible subjects at each site. Accounting for all study locations, 11,544 people were identified, 4325 were enrolled, and 3741 were surveyed. At baseline, 3601 people were available with complete questionnaires. Regarding the follow-up rounds, 2892 (80.3%) individuals were recontacted in the first follow-up, while in the second follow-up, this number was 2726 (75.7%) people. Because this work focused on incident diabetes, prevalent cases were excluded.

## 3. Variables

### 3.1. Outcome Variables

The outcome of interest was incident T2DM. T2DM new cases were identified at the second follow-up because of data availability. Having excluded T2DM cases at baseline (self-reported T2DM diagnosis made by a physician, currently taking medication for diabetes, and a fasting plasma glucose ≥ 126 mg/dl), incident cases were identified if they were receiving medication for T2DM or had a fasting plasma glucose ≥ 126 mg/dl. Blood samples were collected by a trained technician. Venous blood was withdrawn after a fasting period of eight to twelve hours. Plasma glucose was measured with an enzymatic colorimetric technique (GOD-PAP; modular P-E/Roche-cobas, Grenzach-Wyhlen, Germany) [13].

### 3.2. Exposure Variable

The exposure of interest was the HOMA-IR measured at baseline. This variable was computed as glucose (mmol/l) times insulin (*μ*U/ml), all divided by 22.5 [(glucose∗insulin)/22.5] [18]. Blood samples for insulin were retrieved following the same procedures as explained above. Serum insulin was assessed using electrochemiluminescence (modular P-E/Roche-cobas) [13].

### 3.3. Other Variables

Other variables were assessed at baseline following standard questionnaires [13]: sex (men and women); age (35–44, 45–54, 55–64, and ≥65 years); education (<7, 7–11, and ≥12 years of education); study site (Lima, Tumbes, urban Puno, and rural Puno); altitude above the sea level (low altitude included Lima and Tumbes and high altitude encompassed Puno); rurality (urban for Lima as well as urban Puno, semiurban for Tumbes, and rural for rural Puno); and asset index, which is a numerical indicator based on household assets (in tertiles). Weight (kg), height (cm), and waist circumference (cm) were measured with calibrated devices with the participants wearing light clothes and shoeless [13]. Hemoglobin A1C (HbA1c) was assessed with high-performance liquid chromatography (D10, Bio-Rad, Munich, Germany) [13]. For descriptive purposes, the HOMA-B was also estimated: (20 × insulin)/(glucose − 3.5) [18]. Follow-up time was estimated as the time difference between baseline and follow-up, except for people who developed T2DM whose follow-up time was deemed to be half of the observed follow-up time difference. This is a conservative approach taking into consideration that T2DM could have appeared anytime between assessments.

## 4. Statistical Analysis

The analyses were conducted with STATA v13.0 (StataCorp, College Station, TX, USA). For descriptive purposes, numerical variables were summarized with means along with standard deviations or medians with the corresponding interquartile range (IQR). Categorical variables were summarized using proportions. To compare numeric variables according to other categorical variables, the Wilcoxon rank-sum and the Kruskal-Wallis rank tests were used. For comparison of categorical variables, the chi-squared test was conducted. To estimate the *p* value for trend, the Cuzick test (*nptrend* command) was used. The receiver operating characteristic (ROC) curve was plotted, and the area under the ROC curve (AUC) was estimated using the *roctab* command. The AUC was compared among study sites, as well as by altitude and rurality using the *roccomp* command. Finally, empirical thresholds for the HOMA-IR to identify incident T2DM case were estimated with the *cutpt* command and the *youden* option; these calculations yielded the Youden's index, sensitivity, specificity, and the AUC for each given threshold. To estimate the 95% confidence interval (95% CI) of each empirical threshold and its corresponding AUC, a postestimation bootstrap technique was followed assuming one thousand replications without replacement. The positive likelihood ratio was estimated as sensitivity over one minus specificity, whereas the negative likelihood ratio was defined as one minus sensitivity over specificity. The negative predictive value was estimated as [sensitivity × prevalence] divided by [sensitivity × prevalence + (1 − specificity) × (1 − prevalence)]; the negative predictive value was computed as [specificity × (1 − prevalence)] divided by [(1 − sensitivity) × prevalence + specificity × (1 − prevalence)]. In order to assess whether the expected (using the HOMA-IR as predictor) and observed number of diabetes cases matched across all doses of the exposure (the HOMA-IR divided in deciles), we used the Hosmer-Lemeshow chi-squared goodness of fit test; high *p* values (*p* > 0.05) mean that the expected and observed numbers of cases are similar; thus, the predictor shows good calibration. To compare the AUC yielded for incident T2DM between the HOMA-IR and HbA1c, the *roccomp* command was used; this tests if the AUC is equal between assessment methods.

## 5. Ethics

The protocol of the CRONICAS Cohort Study was reviewed and approved by three independent Institutional Review Boards in Peru and in the USA: Universidad Peruana Cayetano Heredia and Associaion Benefica PRISMA in Lima, Peru, as well as by the Johns Hopkins University in Baltimore, USA. All participants gave verbal informed consent [13]. The research was conducted in accordance with the Helsinki declaration.

## 6. Results

### 6.1. Study Population at Baseline

There were 3601 subjects, though 481 (13.4%) were excluded because of missing values in baseline glucose or insulin; there were no differences in gender and age, but excluded subjects were poorer and there were more excluded subjects in Puno (Supplementary Table 1).

At baseline, mean age was 55.6 (SD: 12.7) years, and 51.2% of the study sample was female. The prevalence of T2DM was 6.9% (95% CI: 6.1%–7.9%). The median HOMA-IR was 1.7 (IQR 1.0–2.9) and the median HOMA-B was 96.4 (IQR 59.5–149.7). Further details about these sociodemographic and clinical variables are shown in Table 1, overall and by study site.

### 6.2. HOMA-IR: Characteristics at Baseline

Differences in the HOMA-IR by sociodemographic and clinical variables are shown in Table 2. Median values of the HOMA-IR varied among urban, semiurban, and rural sites: 1.8, 2.0, and 1.1 (*p* for trend < 0.001), respectively. The higher the wealth index, the higher the HOMA-IR: median of 1.4 at the bottom tertile and of 2.0 at the top tertile (*p* for trend < 0.001).

### 6.3. New Diabetes Cases

The mean follow-up time was 2.3 (SD: 0.5) years. Among those who did not have T2DM at baseline, 120 people developed T2DM. Subjects who developed T2DM at follow-up had higher HOMA-IR at baseline in comparison to those who did not develop T2DM (*p* < 0.001): the median was 2.9 (IQR: 1.7–4.3) and 1.7 (IQR: 1.0–2.7), respectively. A similar difference was retrieved when comparing these populations regarding fasting glucose at baseline (*p* < 0.001): subjects who did not develop T2DM had a median glucose of 91.0 mg/dl (IQR: 85.0–97.9) and those who developed T2DM had a median glucose of 102.5 mg/dl (IQR: 91.7–116.0).

### 6.4. HOMA-IR: Diagnostic Performance for Incident Cases

**Figure 1:**
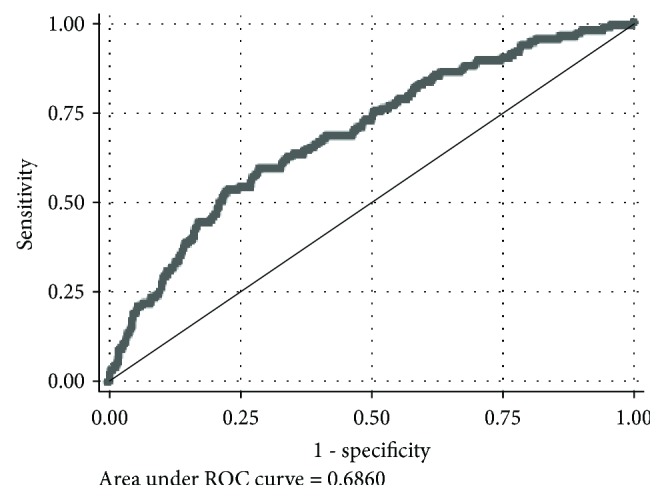
ROC for the HOMA-IR and incident diabetes cases at follow-up.

The AUC for incident T2DM cases was 0.68 (95% CI: 0.64–0.74, Figure 1). The AUC for each study population is presented in Table 3, along with the corresponding empirical thresholds.

**Figure 2:**
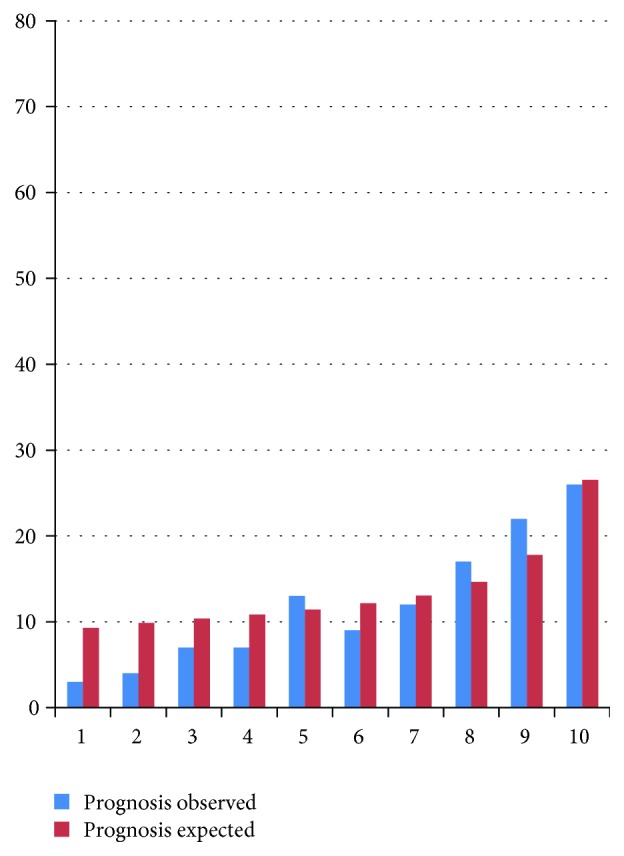
Agreement between expected and observed incident diabetes cases.

Empirical HOMA-IR thresholds were different among study sites, varying from 0.9 (95% CI: 0.00–1.96) to 2.9 (95% CI: 2.11–3.69) in urban Puno and Lima, respectively. Dissimilar thresholds were also found according to altitude: 2.8 (95% CI: 2.20–3.48) at low and 1.2 (95% CI: 0.62–1.71) at high altitude, as well as according to rurality: 2.5 (95% CI: 1.67–3.26) in urban, 2.8 (95% CI: 1.80–3.88) in semiurban, and 1.2 (95% CI: 0.76–1.58) in rural areas. There was evidence of moderate calibration (*p* = 0.065), with good agreement (Figure 2) in the tenth decile (i.e., highest HOMA-IR level).

### 6.5. HOMA-IR in Comparison to HbA1c

The AUC was larger for HbA1c to predict new T2DM cases: 0.75 (95% CI: 0.70–0.80, *p* = 0.023) for HbA1c.

## 7. Discussion

### 7.1. Main Results

In a population-based study conducted in four Peruvian areas at different stages of urbanization as well as with different sociodemographic and health profiles, the HOMA-IR had moderate prediction accuracy for incident T2DM cases in terms of discrimination and calibration. The HOMA-IR thresholds for incident T2DM cases varied according to study site, altitude, and degree of urbanization. The HOMA-IR AUC was smaller in comparison to HbA1c, suggesting that the latter could be preferred to identify people at high risk of T2DM.

### 7.2. Comparison with Other Studies

Our AUC for incident T2DM was moderate, which could signal that these people are still on their way to T2DM, i.e., would develop T2DM in more than 2.3 years of follow-up. Another study with a median follow-up of nine years and using oral glucose tolerance test (OGTT) as the gold standard reported an AUC of 72% for women and 66% for men [7]. Although prospective evidence on the prediction accuracy of the HOMA-IR is still scarce and deserves further comprehensive scrutiny across populations and health backgrounds, the HOMA-IR appears to have a moderate performance to identify incident T2DM cases even after several years.

### 7.3. Result Interpretation

Including all the study population, the threshold for incident T2DM cases was 2.8. Other authors have suggested cutoffs of around 2.2 [7] and 2.0 [10]. These differences could be because of other determinants of insulin resistance, e.g., visceral fat, or because those populations have different T2DM profiles or are at an advanced stage of the nutritional transition. This would mean that populations with higher levels of these risk factors would experience detrimental effects at lower thresholds of insulin resistance [7]. Nevertheless, the distribution of body mass index and waist circumference was comparable among our study population and previous reports, suggesting that other variables would explain these differences, perhaps genetics or other unmeasured anthropometric features, such as skinfolds [7, 10, 19].

According to study site, the 95% CIs of some empirical thresholds overlap, especially the ones from sites in Puno and those of Lima and Tumbes. However, the results do not support using one threshold across all sites. Furthermore, the positive and negative likelihood ratios are also more similar between Lima and Tumbes and between Puno sites. These pairwise similarities could be explained by shared health profiles [4, 20]. This pattern across study sites is also reflected in the empirical thresholds and their 95% CIs according to rurality level: urban and semiurban sites showed 95% CIs that closely overlap, whereas rural sites have different ranges. The evidence when looking at altitude above the sea level appears to be more conclusive and suggests that Puno (i.e., high-altitude sites) needs a different threshold than Lima and Tumbes.

Across sites and empirical thresholds, the negative predictive values were above 95%, suggesting that this test could be used to “rule out” future cases or at least to signal people that could have a less thorough follow-up with other diabetes biomarkers in the near future. Even though all positive likelihood ratio estimates were small and would not suppose a large change in the pretest probability, the largest estimate was found in Tumbes. This could highlight the unhealthy profile of this population [4, 20], thus the already high probability of finding diabetes cases among them. On the other hand, and consistent with the high negative predictive values, there were relevant negative likelihood ratio estimates in Puno. In other words, and consistent with an overall healthier profile of this population, someone with a negative test result would have low probabilities of becoming diabetic in the near future.

### 7.4. Public Health Relevance

Current diabetes guidelines recommend screening for all people above 45 years of age or if they have risk factors for diabetes and to repeat the screening within the next three years, though this could be shorter depending on initial results [21]. Our findings could support testing a person with a negative HOMA-IR result in more than a year, but less than 2.3 years (follow-up period). Our results indicated large negative likelihood ratio and negative predictive value estimates across sites. These metrics suggest that it is unlikely for a person with a negative result to develop T2DM in the next 2.3 years, albeit this evidence should be interpreted in light with the study limitations and according to each patient's characteristics as well as the epidemiological profile of the population. Future studies should verify our results and test their implementation in clinical practice.

Current massive testing with the HOMA-IR could be limited by economic shortcuts, which in turn reflects a slightly less easy laboratory process. In a Peruvian public hospital, a fasting glucose, an OGTT, and an insulin test cost 1.0 US$, 2.8 US$, and 3.4 US$, respectively [22]. In a thirty-month period, a given subject could have three fasting glucose tests, totaling 3.0 US$. If, however, this subject were tested with the HOMA-IR (4.4 US$), he could have undergone one test only in the same period. This may not be cost-effective (3.0 US$ vs. 4.4 US$), also when compared to HbA1c, which yielded better AUC in our results. Nevertheless, the HOMA-IR could be useful in rural areas where laboratories are not available and thus people have additional expenses (e.g., time and transportation) to get screening; thereby, people from rural areas could commute (although this consumes additional time and costs), have a HOMA-IR test, and should it be a negative result, then follow-up evaluations could be delayed one or two years, yet no further than 2.3 years. Our result yielded strong negative predictive values, suggesting that a person with a negative result has low chances of developing T2DM; hence, follow-up visits could be less often than on a yearly basis, as long as the overall health profile allows. These are speculative arguments, and the HOMA-IR requires further studies before its enactment is strongly suggested or discouraged.

Other blood-based test is HbA1c, which, in comparison to the HOMA-IR, appears to be more accurate, cheaper, and less cumbersome because it does not require a fasting period. Notwithstanding, HbA1c needs high-quality processing to secure consistent results and could also have some limitations at high altitude [23]. Other studies have assessed HbA1c to predict T2DM cases. In China, for example, the AUC was 64.5% (medial follow-up: 4.6) [24], while in rural Korea, it was 74% (median follow-up: 4.0 years) [25]. Our AUC for HbA1c (and AUC) for incident T2DM cases was in between these figures.

On the other hand, the overall results at the proposed threshold signal a higher specificity than sensitivity. This means that it would be more likely to identify healthy people (i.e., without risk of developing T2DM). This has public health relevance because knowing who is not likely to develop T2DM would allow allocating (limited) resources to high-risk individuals. Furthermore, the ratio between true positive and false negative, as derived from the overall sensitivity and specificity estimates, is 1.13. This reveals that for each 10 false negatives, roughly 11 true positive cases would be identified.

### 7.5. Strengths and Limitations

This study benefits from a relatively large sample size, but most importantly from including participants from study sites with different sociodemographic and health characteristics. We analyzed prospective data, which allowed us to study the short-term effect of the HOMA-IR to identify new T2DM cases. Nevertheless, limitations of the study need to be acknowledged too. First, our T2DM diagnosis was based on fasting glucose. If in addition to or in replace of this method we have used another one, namely, OGTT, we could have detected more T2DM cases. However, if this were the case, our results are still conservative and there could have been a larger AUC. Second, most of the subjects excluded from the analysis were from Puno. Results for this site should thus be interpreted cautiously. Third, some subjects were lost to follow-up; thus, there could have been more new cases. Our results warrant further verification, i.e., external validation. Fourth, although differences across sites could be attributed to different laboratory methods, all samples were analyzed by the same laboratory minimizing this potential bias.

## 8. Conclusions

Using the HOMA-IR to identify incident T2DM cases seems to yield moderate accuracy. Given the increasing burden of T2DM, actions need to be taken to improve the early identification of these patients. Using available laboratory facilities to assess the HOMA-IR could have positive results.

## Figures and Tables

**Table 1:** Sociodemographic and clinical characteristics of study participants at baseline and overall and according to study site.

Variable	Overall	Lima	Urban Puno	Rural Puno	Tumbes	*p* value
Sex	*N* = 3118	*N* = 1031	*N* = 517	*N* = 539	*N* = 1031	0.882
Female	51.2	51.8	50.9	52.1	50.3	
Male	48.8	48.2	49.1	47.9	49.7	
Age (years)	*N* = 3118	*N* = 1031	*N* = 517	*N* = 539	*N* = 1031	0.934
35–44	24.3	24.0	24.8	22.8	25.3	
45–54	25.5	27.1	25.0	25.4	24.4	
55–64	25.4	25.2	25.7	25.4	25.3	
65+	24.8	23.8	24.6	26.4	25.0	
Wealth index	*N* = 3120	*N* = 1031	*N* = 517	*N* = 541	*N* = 1031	<0.001
Bottom	31.6	12.0	23.2	71.5	34.5	
Middle	33.9	37.0	26.1	26.1	39.0	
Top	34.4	51.0	50.7	2.4	26.5	
Education	*N* = 3118	*N* = 1030	*N* = 517	*N* = 541	*N* = 1030	<0.001
<7 years	45.9	42.8	14.7	62.9	55.6	
7–11 years	32.9	39.7	27.3	30.5	30.3	
≥12 years	21.2	17.5	58.0	6.7	14.1	
Waist circumference	*N* = 3115	*N* = 1031	*N* = 514	*N* = 540	*N* = 1030	<0.001
Mean (SD)	91.9 (11.0)	92.4 (10.5)	92.6 (10.8)	85.0 (11.0)	94.6 (10.2)	
Body mass index	*N* = 3116	*N* = 1031	*N* = 514	*N* = 540	*N* = 1031	<0.001
Mean (SD)	27.7 (4.6)	28.4 (4.6)	27.8 (4.3)	25.1 (3.7)	28.3 (4.8)	
HbA1c	*N* = 3119	*N* = 1031	*N* = 516	*N* = 541	*N* = 1031	
Median (IQR)	5.8 (5.5–6.1)	5.7 (5.4–5.9)	5.9 (5.6–6.1)	5.8 (5.6–6.1)	5.9 (5.6–6.2)	<0.001^∗^
Diabetes	*N* = 3120	*N* = 1031	*N* = 517	*N* = 541	*N* = 1031	<0.001
Yes	6.9	5.5	7.0	3.1	10.3	
HOMA-B	*N* = 3118	*N* = 1030	*N* = 517	*N* = 540	*N* = 1031	<0.001^∗^
Median (IQR)	96.4 (59.5–149.7)	108.0 (68.8–162.8)	95.5 (60.9–153.2)	74.1 (46.1–119.2)	98.6 (57.9–149.2)	
HOMA-IR	*N* = 3120	*N* = 1031	*N* = 517	*N* = 541	*N* = 1031	
Median (IQR)	1.7 (1.04–2.94)	2.0 (1.17–3.19)	1.6 (1.04–2.75)	1.1 (0.62–1.76)	2.0 (1.23–3.42)	<0.001^∗^

Results are presented as percentages for categorical variables. *p* values for categorical variables refer to the chi-squared test among study sites, whereas for numerical variables (waist circumference and body mass index) to the analysis of variance. ^∗^ *p* value refers to the Kruskal-Wallis equality-of-populations rank test among study sites.

**Table 2:** The baseline HOMA-IR according to sociodemographic variables.

Variable	Median (IQR)	*p* value
Sex		<0.001
Female	2.0 (1.2–3.4)	
Male	1.5 (0.9–2.5)	
Age (years)		<0.001/<0.001
35–44	1.8 (1.1–2.9)	
45–54	1.8 (1.1–3.1)	
55–64	1.9 (1.2–3.3)	
65+	1.5 (0.8–2.6)	
Asset index		<0.001/<0.001
Bottom	1.4 (0.8–2.4)	
Middle	1.8 (1.1–3.0)	
Top	2.0 (1.3–3.4)	
Education		<0.001/<0.001
<7 years	1.7 (1.0–2.9)	
7–11 years	1.7 (1.0–2.9)	
≥12 years	2.0 (1.2–3.1)	
Study site		<0.001
Lima	2.0 (1.2–3.2)	
Urban Puno	1.6 (1.0–2.8)	
Rural Puno	1.1 (0.6–1.8)	
Tumbes	2.0 (1.2–3.4)	
Altitude		<0.001/<0.001
Low	2.0 (1.2–3.4)	
High	1.3 (0.8–2.2)	
Rurality		<0.001/<0.001
Urban	1.8 (1.1–3.0)	
Semiurban	2.0 (1.2–3.4)	
Rural	1.1 (0.6–1.8)	
Diabetes		<0.001
No	1.6 (1.0–2.7)	
Yes	4.8 (2.6–8.5)	

Results are presented as median (IQR). *p* values refer to the Wilcoxon rank-sum or to the Kruskal-Wallis tests for independence; if there is a second *p* value, it corresponds to the *p* value for trend.

**Table 3:** Area under the receiver operating characteristic curve for new diabetes cases.

Population	AUC (95% CI)	Empirical cutoff point estimation								
		Empirical threshold (95% CI)	Youden's index	Sensitivity	Specificity	AUC (95% CI)	+LR	−LR	PPV	NPV
Overall	0.6860 (0.6366–0.7355)	2.8 (2.1337–3.5417)	0.31	0.53	0.77	0.65 (0.6099–0.6962)	2.30	0.61	14.59	95.67
Study site										
Lima	0.7114 (0.6294–0.7933)	2.9 (2.1050–3.6879)	0.38	0.62	0.75	0.69 (0.6225–0.7546)	2.48	0.51	12.68	97.12
Urban Puno	0.6233 (0.5171–0.7295)	0.9 (0.0000–1.9610)	0.23	1.00	0.23	0.62 (0.5578–0.6746)	1.30	0.00	8.78	100
Rural Puno	0.6218 (0.4885–0.7550)	1.2 (0.7160–1.6160)	0.36	0.82	0.54	0.68 (0.5687–0.7871)	1.78	0.33	5.46	98.93
Tumbes	0.7281 (0.6464–0.8098)	2.8 (1.8282–3.8472)	0.40	0.67	0.74	0.70 (0.6356–0.7674)	2.58	0.45	22.80	95.14
Altitude above the sea level										
Low	0.7191 (0.6614–0.7768)	2.8 (2.1979–3.4774)	0.39	0.64	0.74	0.69 (0.6460–0.7402)	2.46	0.49	17.43	95.99
High	0.6335 (0.5531–0.7139)	1.2 (0.6240–1.7129)	0.27	0.85	0.42	0.63 (0.5842–0.6848)	1.47	0.36	7.17	98.15
Rurality										
Urban	0.6752 (0.6081–0.7424)	2.5 (1.6665–3.2595)	0.30	0.60	0.70	0.65 (0.5919–0.7088)	2.00	0.57	11.34	96.47
Semiurban	0.7281 (0.6464–0.8098)	2.8 (1.7997–3.8757)	0.40	0.67	0.74	0.70 (0.6355–0.7674)	2.58	0.45	22.80	95.14
Rural	0.6218 (0.4885–0.7550)	1.2 (0.7569–1.5751)	0.36	0.82	0.54	0.68 (0.5757–0.7801)	1.78	0.33	5.46	98.93

*p* values for equality between populations in terms of their areas under the receiver operating characteristic curve were 0.306, 0.090, and 0.366 for study site, altitude, and rurality, respectively; the null hypothesis is that all areas are equal. +LR refers to the positive likelihood ration, whereas −LR refers to the negative likelihood ratio. PPV and NPV refer to positive and negative predictive values, respectively (results presented in %); these were computed with specific prevalence estimates according to the level of the variable (e.g., diabetes prevalence at low altitude).

## Data Availability

The data used to support the findings of this study are available from the corresponding author upon request.

## Supplementary Materials

Supplementary Materials Table S1: comparison between included and excluded subjects in the analysis.
