# Supplementary material for: The HOMA-IR Performance to Identify New Diabetes Cases by Degree of Urbanization and Altitude in Peru: The CRONICAS Cohort Study
Source: J Diabetes Res. 2018 Dec 16;2018:7434918. doi: 10.1155/2018/7434918 (PMC6311843; doi:10.1155/2018/7434918)
Supplement: Supplementary Materials — Table S1: comparison between included and excluded subjects in the analysis. [file 7434918.f1.docx]

Supplementary Table 1: Comparison between included and excluded subjects in the analysis.
